# Lithotomy in an Adult under the Influence of Chloroform

**Published:** 1849-05

**Authors:** F. H. Hamilton


ART. V. — *Lithotomy in an Adult under the Influence of Chloroform.* By F. H. Hamilton, M. D.

James Martin, aged 24, entered the "Buffalo Hospital of the Sisters of Charity," February 20th, 1849.

Martin was born in Nunda, Allegany Co., in this state, and moved from there, when he was ten years old, to Evans, Erie Co., where he has resided until the present time. From infancy he has been afflicted with enuresis. Six years since, he first had indications of the existence of a calculus in his bladder. His health is considerably impaired.

During three days, he was subjected to an absolute diet, and bowels gently cathartacised.

February 24th. The operation was made in presence of the Hospital class. Patient was tolerably, but not completely narcotized by chloroform. The incisions were made by the lateralized mode, with a common bistoury, and completed after reaching the urethra, with a straight, broad pointed, and narrow bistoury. The stone was found so large, that I was compelled, after ineffectual efforts to crush it, to enlarge the incision through the prostate, after which, it was removed with a moderate traction. Weight of calculus, a fraction over four ounces. Longest diameter, two inches and seven eighths. Longest transverse diameter, two inches. Chemical character, ammonio-phosphatic. No arteries required the ligature, and no accident complicated the operation. The forefinger of the left hand was kept in the rectum, and the thumb followed the wound until the urethra was reached, as in the operation upon the lad. I still think it a valuable modification of the usual mode of procedure.

Patient continued to do well, with the exception of having had an occasional diarrhœa, and an attack of erysipelas on the nose and face, and a slight tenderness of abdomen, all of which yielded readily to treatment, until the 9th of March, two weeks after the operation was made. He died suddenly, in the night of the 9th, without any premonition of the event.

*Autopsy.*—Bladder thickened, and free from matter or fragments of stone. Peritoneum in lower part of abdomen, covered with lymph, and adherent. Pus underneath the peritoneum, in the loose cellular texture, in every direction around the bladder, but especially abundant below, surrounding the rectum, and forming a considerable depot against the sacrum. No other morbid conditions. I ascribe his death to purulent absorption.

